# Salivary Testosterone Levels and Health Status in Men and Women in the British General Population: Findings from the Third National Survey of Sexual Attitudes and Lifestyles (Natsal-3)

**DOI:** 10.1210/jc.2016-1669

**Published:** 2016-08-23

**Authors:** S. Clifton, W. Macdowall, A. J. Copas, C. Tanton, B. G. Keevil, D. M. Lee, K. R. Mitchell, N. Field, P. Sonnenberg, J. Bancroft, C. H. Mercer, A. M. Wallace, A. M. Johnson, K. Wellings, F. C. W. Wu

**Affiliations:** Research Department of Infection and Population Health (S.C., A.J.C., C.T., N.F., P.S., C.H.M., A.M.J.), University College London, London WC1E 6BT, United Kingdom; Department of Social and Environmental Health Research (W.M., K.R.M., K.W.), London School of Hygiene and Tropical Medicine, London WC1E 7HT, United Kingdom; Department of Clinical Biochemistry (B.G.K.), University Hospital S Manchester, Manchester M13 9WL, United Kingdom; Cathie Marsh Institute for Social Research, School of Social Sciences (D.M.L.), The University of Manchester, Manchester M13 9WL, United Kingdom; MRC/CSO Social and Public Health Sciences Unit (K.R.M.), University of Glasgow, Glasgow G12 8QQ, United Kingdom; Kinsey Institute (J.B.), Indiana University, Bloomington, Indiana 47405; Department of Clinical Biochemistry (A.M.W.), Royal Infirmary, Glasgow G4 0SF, United Kingdom; and Andrology Research Unit (F.C.W.W.), Manchester Centre of Endocrinology and Diabetes, Manchester Academic Health Science Centre, The University of Manchester, Manchester M13 9PL, United Kingdom

## Abstract

**Context:**

Salivary T (Sal-T) measurement by liquid chromatography–tandem mass spectroscopy resents the opportunity to examine health correlates of Sal-T in a large-scale population survey.

**Objective:**

This study sought to examine associations between Sal-T and health-related factors in men and women age 18–74 years.

**Design and Setting:**

Morning saliva samples were obtained from participants in a cross-sectional probability-sample survey of the general British population (Natsal-3). Self-reported health and lifestyle questions were administered as part of a wider sexual health interview.

**Participants:**

Study participants included 1599 men and 2123 women.

**Methods:**

Sal-T was measured using liquid chromatography–tandem mass spectroscopy. Linear regression was used to examine associations between health factors and mean Sal-T.

**Results:**

In men, mean Sal-T was associated with a range of health factors after age adjustment, and showed a strong independent negative association with body mass index (BMI) in multivariable analysis. Men reporting cardiovascular disease or currently taking medication for depression had lower age-adjusted Sal-T, although there was no association with cardiovascular disease after adjustment for BMI. The decline in Sal-T with increasing age remained after adjustment for health-related factors. In women, Sal-T declined with increasing age; however, there were no age-independent associations with health-related factors or specific heath conditions with the exception of higher Sal-T in smokers.

**Conclusions:**

Sal-T levels were associated, independently of age, with a range of self-reported health markers, particularly BMI, in men but not women. The findings support the view that there is an age-related decline in Sal-T in men and women, which cannot be explained by an increase in ill health. Our results demonstrate the potential of Sal-T as a convenient measure of tissue androgen exposure for population research.

## Abstract (precis)

Sal-T levels in a general population study were associated with BMI in men. An agerelated decline in Sal-T in men and women was found which cannot be explained by an increase in ill-health

In men, T deficiency with pituitary or testicular disease (male hypogonadism) is known to cause a wide range of symptoms and conditions including sexual dysfunction, loss of vigor, poor physical performance, fractures, and low mood, which can be effectively treated with testosterone replacement therapy (TRT) (1). However, the more general relationship between male T levels and ill-health is less well understood. Community-based studies in men have shown associations between lower average serum testosterone (serum-T) and poorer health outcomes, including frailty, obesity, insulin resistance, cardiovascular diseases, and mortality, although findings have not always been consistent (2–5). It is suggested that lower T levels in older men may contribute to these ageing-related conditions although questions remain about direction and causality (3). Some studies have also cast doubt as to whether there truly is an independent age-related decrease in T, or whether the observed decline is a biomarker of accumulated comorbidities (6). Currently, whether T treatment would benefit symptomatic older men with low T remains a subject of intense debate and research (7, 8). Many of the existing studies have been carried out in middle-age or older men, and the health implications of lower T in younger men are unclear. In women, circulating concentrations of T are typically around 5–10% of those in men, with distinct age-related declines, independent of the menopause (9, 10). The evidence for associations between T and health in women is even more equivocal than for men (10–13). Furthermore, research efforts in women have been seriously hampered by inadequate sensitivity of serum-T measurements, due to the low concentration of T and poor specificity of commonly used immunoassay methods (14).

Salivary testosterone (Sal-T) is believed to represent tissue hormone levels, which are unaffected by variations in circulating binding proteins (15), thereby providing an alternative to serum free testosterone (free-T) in the assessment of androgen status. In contrast to the collection of serum, which is invasive and expensive, collection of saliva is relatively straightforward and requires minimal training. We have recently demonstrated that Sal-T can be reliably and accurately measured by a highly sensitive and specific liquid chromatography–tandem mass spectrometry method (16). In a validation study comparing samples from the same individuals, we found that Sal-T in adult men and women correlated more strongly with calculated serum free-T than serum total-T (17) and was also unaffected by variations in sex hormone-binding globulin (SHBG) (18). Fiers at al (19) confirmed the good correlation in both men and women between Sal-T and serum free-T measured by equilibrium dialysis but there was a significant systematic positive bias in women, which may reflect the influence of salivary protein binding on the lower female concentrations of Sal-T. The physiological and health-related behavioral correlates of Sal-T have not yet been explored.

Using data from the third National Survey of Sexual Attitudes and Lifestyles (Natsal-3), a probability-sample survey of British men and women, we investigated whether Sal-T is associated, independently of age, with demographic characteristics, lifestyle, general health, and reported health conditions. We hypothesized that relationships between Sal-T and health-related factors in men would be similar to those previously observed with serum free-T. To our knowledge, this is the first study to have examined the associations between Sal-T and health in a large community sample of men and women using a highly sensitive and specific assay, exploiting the theoretical and practical advantages of salivary measurements to the full.

## Materials and Methods

### Participants and procedures

Full details of the Natsal-3 methods, including details of the saliva sample collection and testing, are described elsewhere (20, 21). Briefly, Natsal-3 was a probability-sample survey of 15 162 men and women age 16–74 years resident in Britain. Interviews took place between September 2010 and August 2012 using a combination of computer-assisted personal interviewing and computer-assisted self-interview for the more-sensitive questions. The response rate was 57.7%.

Single morning saliva samples were self collected from a subsample of men and women age 18–74 years who did not regularly work night shifts. Consenting participants were given a self-collection pack and asked to provide their sample before 10:00 hours, to minimize diurnal variation in T (7). They were asked not to brush their teeth, eat, or chew before giving the sample, and to spit directly into a plain polystyrene tube. Saliva samples were posted to the laboratory where they were prepared and frozen at −80°C until analysis. On receipt of the sample, participants were sent a £5 voucher as a token of appreciation. Altogether, 9170 eligible participants were invited to provide a saliva sample: 6515 (71.0%) agreed to do so and 4591 samples were received by the laboratory and matched to the survey data (50.1% of those invited). A total of 463 samples were excluded due to sample quality issues (insufficient volume [n = 154]; sample discolored/bloody [n = 91]; sample recorded as taken after 10:30 hours [n = 34]; period between sample being taken and received by the laboratory more than 5 days or unknown due to missing date of collection [n = 172]; not tested due to error [n = 12]) leaving 4128 participants (45.0% of those invited) with a T result (1675 men; 2453 women). This paper examines associations between Sal-T and health in the general population; therefore, 76 men and 330 women with clinical conditions or taking medication likely to affect T levels were excluded from analysis (individuals may be excluded for more than one reason): currently taking medication for epilepsy (15 men; 15 women) or prostate disease (43 men); treatment for ovarian, testicular, or pituitary condition (16 men; 23 women) or for polycystic ovaries (35 women) in the past year; pregnant at interview (42 women); current receipt of hormone replacement therapy (62 women); ever receipt of hormone replacement therapy together with having had a hysterectomy (proxy measure for having had ovaries removed; 181 women); missing data for these questions (three men; 15 women); resulting in 1599 men and 2123 women being included in the analysis. These exclusions aimed to minimize confounding of the relationship between T and health caused by these factors which are known to influence T levels, while retaining an otherwise-representative sample of the general population. Women taking hormonal contraception (HC) (oral contraceptive pill, mirena, injections, implants, or the contraceptive patch) were included in analyses to allow examination of the relationship between HC and sal-T, and to avoid biases which may result from excluding this substantial proportion of women (24% of all women with a valid saliva sample usually used a HC, and this was 67% in the youngest age group [age 18–24 y]). However, additional sensitivity analyses were carried out excluding women taking HC, to assess whether their inclusion affected associations with health factors.

### Measures

Health and medication questions were self reported, and body mass index (BMI) was calculated from self-reported height and weight. Due to small numbers of underweight individuals (BMI < 18.5 kg/m^2^), these participants were excluded from analysis of BMI (14 men and 44 women). Most of the health questions were asked in the computer-assisted personal interviewing, with the exception of depressive symptoms (past 2 weeks) which were assessed in the computer-assisted self interview using a validated two-item patient health questionnaire (PHQ-2) (22). Cardiac, vascular disease, or hypertension were defined as ever being given such diagnoses by a doctor.

Season of data collection was included as a potential confounder, defined as: Winter (December, January, February), Spring (March, April, May), Summer (June, July, August), Autumn (September, October, November).

The liquid chromatography–tandem mass spectroscopy Sal-T assay was developed using strict validation criteria (23), with a lower limit of quantification of 6.5 pmol/L. Full details of the laboratory methods, including the validation of the assay, have been published elsewhere (17, 18, 20).

### Statistical analyses

Statistical analyses were carried out using STATA (version 13.1) accounting for the complex survey design (stratification, clustering, and weighting of the sample) (24). We applied two weights: the survey weight corrected for unequal probability of selection and differential response (by age, sex, and region) to the survey itself; the additional saliva weight corrected for unequal probability of selection and differential response to the saliva sample. Factors found to be associated with providing a usable saliva sample included age at interview, ethnicity, self-reported general health, and sexual function; the saliva weighting reduced these biases (20).

Descriptive statistics are presented as means (SEs), with multivariable linear regression used to assess differences between groups. Throughout, we censored very high Sal-T values so that, for each 10-year age group stratified by sex, values above the 99th percentile were assigned a value equal to that of the 99th percentile. The Sal-T data for men were normally distributed; however, the distribution for women was positively skewed and so values were transformed on the natural log scale for analysis. Accordingly, for men we present linear regression coefficients representing differences in mean T in pmol/L, whereas for women we present ratios of geometric mean Sal-T obtained from exponentiated coefficients. Interval regression was used to assign values to the range 0 to 6.5 pmol/L for three men, and 0.5 (to allow log transformation) to 6.5 pmol/L for 62 women with T levels below the limit of detection (<6.5 pmol/L). Age was adjusted for using both linear and quadratic terms to account for a nonlinear relationship of T with age.

Given that several associations were found for men, multivariable analyses were used to determine which health factors were independently associated with Sal-T. Variables were grouped into a series of domains, to identify their individual contributions to the overall relationships with Sal-T after adjustment for earlier domains. The domains were: 1) age and season, 2) relationship status and children, 3) BMI, and 4) general health. A fifth lifestyle domain (smoking, alcohol consumption, drug use) contained no significant associations (either age adjusted or multivariable) and was therefore not presented in the final multivariable table. Within each domain the variables were entered into a forward stepwise model selection process (significance level for inclusion *P* < .1) with variables selected from earlier domains included with certainty. The ordering of domains began with factors for which the evidence of association was best established (from earlier publications) or of a demographic nature, and then proceeded to health-related factors. In this way, any identified associations between Sal-T and health could be seen as robust and not explained by confounding factors in other domains. An equivalent analysis was not performed for women given the lack of age-adjusted associations. In further analysis we examined associations between specific health conditions or medical treatments and Sal-T, adjusting firstly for age and then for those factors that had been cumulatively selected from domains 1 to 3.

### Ethics

The Natsal-3 study was approved by the Oxfordshire Research Ethics Committee A (reference: 09/H0604/27). Written informed consent was obtained for anonymized testing of saliva samples, without return of results.

## Results

### Age-adjusted associations of demographic and general health factors with mean Sal-T (Tables 1 and 2; Figures 1 and 2)

**Table 1.** Sample Characteristics, Mean Sal-T, and Associations Between Sal-T and Demographic and Health Factors: Men

Characteristics	Denominators, n		se	Mean Sal-T, pmol/L	Age-Adjusted^c^ Coefficients	95% CI
	unwt	wt				
All men	1599	1886	3.33	223.49		
Age, season, region						
Age group					*P* < .0001	
18–24 y	186	242	9.14	315.81	1.00	—
25–34, y	245	328	7.77	264.62	−51.19	(−74.44, −27.94)
35–44, y	236	363	8.42	234.16	−81.65	(−105.90, −57.41)
45–54, y	297	388	5.11	205.74	−110.08	(−130.78, −89.37)
55–64, y	330	334	4.34	174.63	−141.18	(−160.84, −121.52)
65–74, y	305	231	3.74	151.72	−164.09	(−183.51, −144.68)
Season					*P* = .0360	
Winter	400	500	6.93	235.15	1.00	—
Spring	464	521	6.09	223.77	−5.75	(−20.90, 9.40)
Summer	421	457	6.02	207.52	−20.73	(−36.74, −4.72)
Autumn	314	408	6.40	226.74	−2.95	(−19.42, 13.52)
Region					*P* = .2432	
Scotland and North of England	558	639	5.74	230.52	1.00	—
Midlands and Wales	355	391	6.48	224.33	−7.20	(−21.22, 6.81)
East of England and South of England	686	856	5.17	217.86	−11.08	(−24.14, 1.99)
Relationship and children						
Relationship status					*P* = .0103	
Married/civil partnership/cohabiting	928	1277	3.95	206.71	1.00	—
Steady relationship, not living together	187	179	8.34	263.63	14.62	(−1.89, 31.13)
Not in a steady relationship	459	405	6.97	261.46	20.12	(6.41, 33.82)
Any natural children					*P* = .374	
No	642	736	5.99	256.27	1.00	—
Yes	928	1104	3.62	204.97	−5.80	(−18.61, 7.00)
Lives with a child (age < 18 y)					*P* = .7611	
No	1281	1342	3.76	220.84	1.00	—
Yes	318	544	6.18	230.05	−2.17	(−16.21, 11.86)
BMI						
BMI^a^					*P* < .0001	
Normal (BMI 18.5–25 kg/m^2^)	599	723	5.07	253.68	1.00	—
Overweight (BMI 25–30 kg/m^2^)	612	715	4.09	209.82	−23.63	(−34.81, −12.46)
Obese (BMI > 30 kg/m^2^)	334	365	5.72	174.34	−51.26	(−64.64, −37.88)
General health and function						
Self-reported general health					*P* = .0054	
Very good/good	1253	1510	3.83	232.72	1.00	—
Fair/bad/very bad	346	375	5.08	186.37	−16.14	(−27.50, −4.78)
Longstanding illness or disability					*P* = .0131	
No	977	1251	4.15	238.63	1.00	—
Yes	621	634	4.54	193.69	−13.36	(−23.91, −2.81)
Difficulty walking up stairs because of a health problem					*P* = .0499	
No difficulty	1369	1643	3.59	230.41	1.00	—
Some difficulty	166	177	7.60	182.11	−8.31	(−23.34, 6.71)
Much difficulty or unable	64	66	12.08	162.09	−23.74	(−44.08, −3.41)
No. of comorbid conditions^b^					*P* = .0073	
0	913	1169	4.38	245.12	1.00	—
1	397	435	5.87	201.40	−11.10	(−24.18, 1.98)
2+	289	282	5.49	167.97	−21.57	(−35.10, −8.04)
Lifestyle						
Current smoker					*P* = .6599	
No	1247	1458	3.60	219.63	1.00	—
Yes	352	428	7.20	236.67	3.01	(−10.42, 16.45)
Average alcohol consumption per wk					*P* = .3611	
None	295	343	7.44	213.51	1.00	—
Not more than recommended	1130	1352	3.98	226.02	9.51	(−5.57, 24.59)
More than recommended	167	183	9.83	223.35	14.50	(−7.32, 36.32)
Taken non-prescribed drugs, past y					*P* = .7584	
No	1363	1585	3.52	218.03	1.00	—
Yes	197	244	9.03	264.34	2.89	(−15.56, 21.34)

Abbreviations: unwt, unweighted; wt, weighted.

a Those with BMI < 18.5 kg/m^2^ have been excluded from analysis due insufficient numbers to analyze this group separately.

b Measure of comorbidity includes arthritis, heart attack, coronary heart disease, angina, other forms of heart disease, hypertension, stroke, diabetes, broken hip or pelvis bone or hip replacement ever, backache lasting longer than 3 months, any other muscle or bone disease lasting longer than 3 months, treatment for depression, treatment for cancer, and treatment for any thyroid condition in the past year.

c Adjusted for age and age squared to account for nonlinear relationship between T and age.

**Table 2.** Sample Characteristics, Mean Sal-T, and Associations Between Sal-T and Demographic and Health Factors: Women

Characteristics	Denominators, n		se	Mean Sal-T, pmol/L	Age-Adjusted Ratios^c^	95% CI
	unwt	wt				
All	2123	1899	0.86	37.09		
Age, season, region						
Age group					*P* < .0001	*P* < .0001
18–24 y	231	252	4.15	49.98	1.00	—
25–34, y	390	359	1.82	41.74	0.93	(0.78, 1.12)
35–44, y	391	381	1.92	40.49	0.91	(0.76, 1.09)
45–54, y	408	390	1.55	34.16	0.76	(0.63, 0.91)
55–64, y	378	304	0.98	27.35	0.63	(0.53, 0.75)
65–74, y	325	213	1.25	27.24	0.60	(0.50, 0.72)
Season					*P* < .0001	
Winter	529	460	1.09	31.57	1.00	—
Spring	652	556	1.95	37.71	1.15	(1.05, 1.26)
Summer	491	394	1.83	42.46	1.28	(1.16, 1.40)
Autumn	451	489	1.65	37.27	1.09	(0.98, 1.20)
Region					*P* = .2229	
Scotland and North of England	741	632	1.19	35.66	1.00	—
Midlands and Wales	475	408	2.19	35.09	0.96	(0.87, 1.05)
East of England and South of England	907	859	1.32	39.10	1.04	(0.96, 1.13)
Relationship and children						
Relationship status					*P* = .9109	
Married/civil partnership/cohabiting	1203	1234	0.92	35.50	1.00	—
Steady relationship, not living together	257	196	2.39	42.54	1.01	(0.89, 1.14)
Not in a steady relationship	646	449	2.08	39.75	1.02	(0.94, 1.10)
Any natural children					*P* = .6694	
No	597	520	2.25	43.25	1.00	—
Yes	1497	1336	0.78	34.67	1.02	(0.93, 1.12)
Lives with a child (age < 18 y)					*P* = .5441	
No	1409	1179	1.15	35.80	1.00	—
Yes	711	717	1.29	39.09	1.03	(0.94, 1.12)
BMI						
BMI^a^					*P* = .7452	
Normal (BMI 18.5–25 kg/m^2^)	965	870	1.23	39.39	1.00	—
Overweight (BMI 25–30 kg/m^2^)	586	504	1.37	34.82	0.97	(0.89, 0.94)
Obese (BMI > 30 kg/m^2^)	428	355	1.53	35.13	0.97	(0.90, 0.94)
General health and function						
Self-reported general health					*P* = .3880	
Very good/good	1753	1560	0.98	37.91	1.00	—
Fair/bad/very bad	370	339	1.65	33.33	0.96	(0.88, 1.05)
Longstanding illness or disability					*P* = .8803	
No	1360	1281	1.11	38.21	1.00	—
Yes	763	618	1.37	34.77	1.01	(0.92, 1.10)
Difficulty walking up stairs because of a health problem					*P* = .4682	
No difficulty	1787	1598	0.95	38.11	1.00	—
Some difficulty	241	221	2.62	32.92	0.99	(0.87, 1.13)
Much difficulty or unable	95	80	2.79	28.4	0.87	(0.69, 1.09)
No. of comorbid conditions^b^					*P* = .5587	
0	1177	1101	1.22	39.52	1.00	—
1	546	474	1.33	35.01	0.99	(0.91, 1.08)
2+	400	324	2.00	31.91	0.93	(0.82, 1.06)
Lifestyle						
Current smoker					*P* = .0165	
No	1714	1548	0.82	35.88	1.00	—
Yes	409	351	2.45	42.44	1.11	(1.02, 1.22)
Average alcohol consumption per wk					*P* = .3272	
None	640	606	1.57	36.04	1.00	—
Not more than recommended	1280	1113	1.13	37.97	1.06	(0.98, 1.16)
More than recommended	198	175	2.08	35.23	1.02	(0.91, 1.15)
Taken non-prescribed drugs, past y					*P* = .3064	
No	1970	1746	0.90	36.68	1.00	—
Yes	115	106	4.01	46.44	1.09	(0.93, 1.28)

Abbreviations: unwt, unweighted; wt, weighted.

a Those with BMI < 18.5 kg/m^2^ have been excluded from analysis due insufficient numbers to analyze this group separately.

b Measure of comorbidity includes arthritis, heart attack, coronary heart disease, angina, other forms of heart disease, hypertension, stroke, diabetes, broken hip or pelvis bone or hip replacement ever, backache lasting longer than 3 mo, any other muscle or bone disease lasting longer than 3 mo, treatment for depression, treatment for cancer, and treatment for any thyroid condition in the past year.

c Ratio of geometric means, obtained from exponentiated age-adjusted linear regression coefficients of log-transformed data for women. Adjusted for age and age-squared to account for nonlinear relationship between T and age.

**Figure 1.**
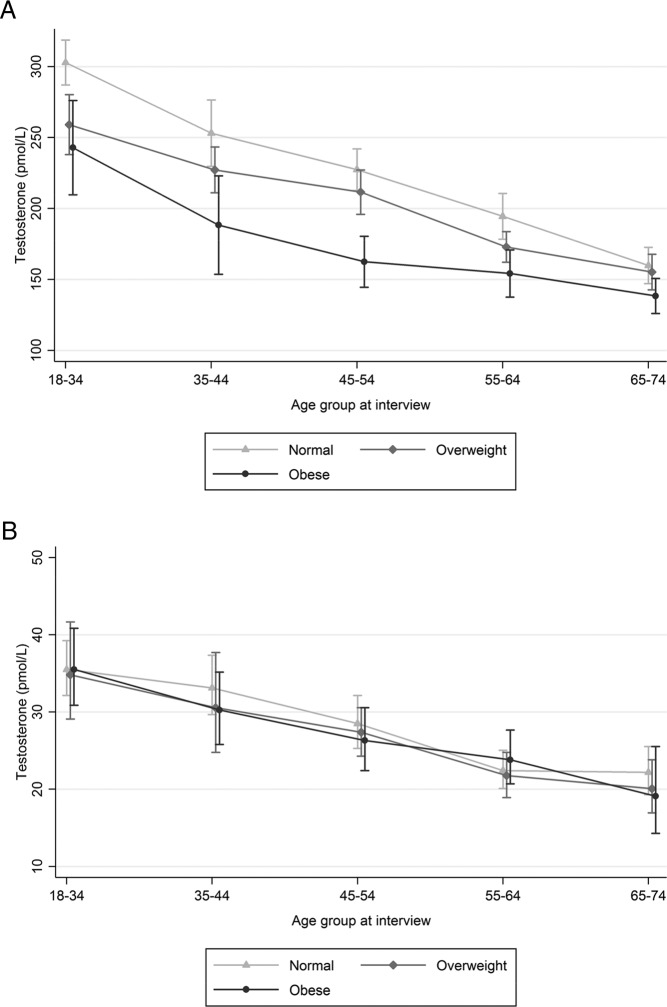
Mean (95% CI) Sal-T (pmol/L) by age group and BMI. Note: figures for men and women have different scales. A, Men. B, Women.

**Figure 2.**
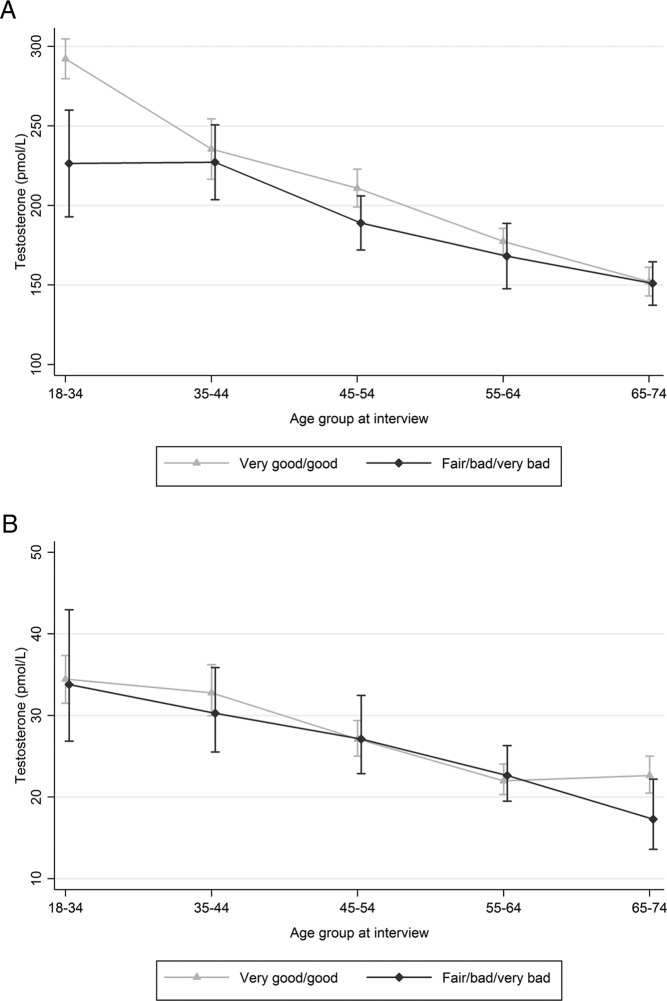
Mean (95% CI) Sal-T (pmol/L) by age group and self-reported general health status. Note: figures for men and women have different scales. A, Men. B, Women.

For both men and women, mean Sal-T decreased with increasing age, and seasonal variation was observed. Among men, there was variation in mean sal-T by relationship status after adjustment for age, with the highest levels among those who were not currently in a steady relationship, and lowest levels among those who were married or cohabiting (age-adjusted linear regression coefficient for those not in a steady relationship, compared with those married/cohabiting: 20.12 (95% confidence interval [CI], 6.41–33.82); no association was found with having children. There were no associations with demographic factors for women.

Mean Sal-T was associated, independently of age, with a range of measures of general health in men but not in women. In age-stratified analyses of men, mean Sal-T was seen to be lower with increasing BMI (normal, overweight, obese) (Figure 1), and somewhat higher in those reporting good or very good health (Figure 2) at least at younger ages; these associations were not seen in women. Expressed as age-adjusted regression coefficients, we found levels were lower among men who were overweight or obese, (coefficients −23.63 [−34.81 to −12.46] and −51.26 [−64.64 to −37.88] respectively, compared with normal BMI) reported poorer general health (−16.14 [−27.50 to −4.78]), longstanding illness or disability (−13.36 [−27.50 to −4.78]); difficulty walking up stairs (−23.74 [−44.08 to −3.41] for those with much difficulty/inability, compared with no difficulty), or two or more comorbid health conditions ([21.57 [−35.10 to −8.04) compared with none). There was no association among men with smoking, alcohol consumption, or illicit drug use.

The only health association found for women was with smoking, with higher Sal-T among women who smoked (ratio of geometric means 1.11 [1.02–1.22] for current smokers compared with nonsmokers). Women who used HC as their usual method of contraception had lower mean Sal-T (geometric mean ratio compared with those who had not taken HC: 0.78 [0.70–0.87]; *P* < .0001). A sensitivity analysis excluding women on HC (n = 499) produced generally similar results, although the association with smoking became nonsignificant due to an increase in the size of the confidence intervals (age-adjusted geometric mean ratio: 1.08 [0.98–1.19]; *P* = .107). Adjusting the women's analysis for season as well as age did not change any of the results (data not shown).

### Multivariable analyses of men

In men, all associations with variables in domains 1–3 in the age-adjusted analyses remained significant in the multivariable analyses: age, season, relationship status, and BMI (Table 3). Only one variable from domain 4—self-reported general health—was selected in the model, although providing inconclusive evidence of an association (−11.39 [−22.69 to −0.10] *P* = .048). In further exploratory analysis we fitted a model excluding BMI but including age, season, and relationship status, in which self-reported general health was found to be more strongly associated with Sal-T (−19.01 [−30.48 to −7.54] *P* = .001; data not shown).

**Table 3.** Adjusted Associations Between Demographic and Health Factors and Salivary Sal-T: Men

Associations	Denominators, n		Age-Adjusted Coefficients	(95% CI)	Domain 1 Adjusted Coefficients	(95% CI)	Domain 2 Adjusted Coefficients	(95% CI)	Domain 3 Adjusted Coefficients	(95% CI)	Domain 4 Adjusted Coefficients	(95% CI)
	unwt	wt										
Age, season, region												
Age				*P* < .0001		*P* < .0001		*P* < .0001		*P* < .0001		*P* < .0001
18–24 y	186	242	1.00	—	—	—	—	—	—	—	—	—
25–34 y	245	328	−51.19	(−74.44, −27.94)	−52.08	(−75.40, −28.75)	−41.05	(−65.54, −16.56)	−33.70	(−58.31, −9.09)	−31.90	(−56.44, −7.35)
35–44 y	236	363	−81.65	(−105.90, −57.41)	−81.66	(−105.55, −57.78)	−69.74	(−94.08, −45.39)	−62.79	(−86.52, −39.07)	−60.66	(−84.61, −36.71)
45–54 y	297	388	−110.08	(−130.78, −89.37)	−109.69	(−130.35, −89.03)	−97.21	(−118.62, −75.81)	−82.80	(−104.26, −61.35)	−79.74	(−101.33, −58 .16)
55–64 y	330	334	−141.18	(−160.84, −121.52)	−140.78	(−160.37, −121.18)	−126.87	(−147.78, −105.96)	−111.77	(−132.95, −90.59)	−107.91	(−128.99, −86.82)
65–74 y	305	231	−164.09	(−183.51, −144.68)	−163.73	(−183.04, −144.42)	−149.80	(−170.91, −128.70)	−134.69	(−155.81, −113.56)	−130.48	(−151.83, −109.13)
Season				*P* = .0360		*P* = .0360		*P* = .0256		*P* = .0368		*P* = .0294
Winter	400	500	1.00	—	—	—	—	—	—	—	—	—
Spring	464	521	−5.75	(−20.90, 9.40)	−5.75	(−20.90, 9.40)	−6.57	(−21.79, 8.66)	−1.93	(−15.68, 11.81)	−2.00	(−15.76, 11.75)
Summer	421	457	−20.73	(−36.74, −4.72)	−20.73	(−36.74, −4.72)	−21.48	(−37.47, −5.49)	−16.41	(−30.21, −2.61)	−16.76	(−30.56, −2.96)
Autumn	314	408	−2.95	(−19.42, 13.52)	−2.95	(−19.42, 13.52)	−2.90	(−19.44, 13.64)	1.24	(−13.03, 15.52)	1.46	(−12.79, 15.71)
Relationship and children												
Relationship status				*P* = .0103				*P* = .0073		*P* = .0185		*P* = .0110
Married/civil partnership/cohabiting	928	1277	1.00	—			—	—	—	—	—	
Steady relationship, not living together	187	179	14.62	(−1.89, 31.13)			16.02	(−0.55, 32.58)	11.18	(−4.54, 26.91)	11.68	(−4.07, 27.42)
Not in a steady relationship	459	405	20.12	(6.41, 33.82)			20.50	(6.85, 34.15)	18.52	(5.13, 31.91)	20.10	(6.50, 33.70)
Any natural children				*P* = .374								
No	642	736	1.00	—								
Yes	928	1104	−5.80	(−18.61, 7.00)								
Lives with a child (age < 18 y)				*P* = .7611								
No	1281	1342	1.00	—								
Yes	318	544	−2.17	(−16.21, 11.86)								
BMI										*P* < .0001		*P* < .0001
BMI				*P* < .0001								
Normal (BMI, 18.5–25 kg/m^2^)	599	723	1.00	—					—	—	—	—
Overweight (BMI, 25–30 kg/m^2^)	612	715	−23.63	(−34.81, −12.46)					−22.40	(−33.63, −11.17)	−22.33	(−33.56, −11.11)
Obese (BMI > 30 kg/m^2^)	334	365	−51.26	(−64.64, −37.88)					−49.69	(−62.84, −36.54)	−47.59	(−61.22, −33.96)
General health and function												
Self-reported general health				*P* = .0054								*P* = .048
Very good/good	1253	1510	1.00	—						—	—	—
Fair/bad/very bad	346	375	−16.14	(−27.50, −4.78)							−11.39	(−22.69, −0.10)
Longstanding illness or disability				*P* = .0131								
No	977	1251	1.00	—								
Yes	621	634	−13.36	(−23.91, −2.81)								
Difficulty walking up stairs because of a health problem				*P* = .0499								
No difficulty	1369	1643	1.00	—								
Some difficulty	166	177	−8.31	(−23.34, 6.71)								
Much difficulty or unable	64	66	−23.74	(−44.08, −3.41)								
Number of comorbid conditions				*P* = .0073								
0	913	1169	1.00	—								
1	397	435	−11.10	(−24.18, 1.98)								
2+	289	282	−21.57	(−35.10, −8.04)								

Abbreviations: unwt, unweighted; wt, weighted.

Variables were grouped into a hierarchy of domains as follows: 1. Age, season, region; 2. Relationship status and family; 3. BMI; 4. General health and function. Lifestyle factors (smoking, drinking, drug use) were not included in the adjusted analysis given lack of associations seen in age-adjusted analysis.

The variables were entered in these groups in order, and including any variables found to be significant from previous domains, into forwards stepwise linear (interval) regression models to generate adjusted coefficients. The significance criteria for retention in the model was *P* < .1.

### Specific health conditions

Cardiovascular disease (including hypertension) was reported by 19% of men in our sample, and was associated with lower mean Sal-T in men, independently of age (−18.06 [−29.43 to −6.68]) (Table 4). There was also an association with currently taking medication for depression (−24.56 [−48.33 to −0.79], reported by 4% of men), but not with current depressive symptoms (10% of men screened positive for depressive symptoms in the past 2 weeks). After adjustment for age, there was at best weak evidence of an association with self-reported doctor-diagnosed diabetes (reported by 6% of men in our sample). Further adjustment for season, relationship status, and BMI attenuated the association with cardiovascular disease (−8.98 [−20.27 to 2.31]; *P* = .119) and diabetes (−5.51 [−23.1 to 12.1]; *P* = .540) but strengthened the association with depression medication (−28.02 [−51.64 to −4.40]; *P* = .020). There was no evidence of associations between Sal-T and any of these conditions among women.

**Table 4.** Associations Between Reporting Specific Health Conditions and Medical Treatments and Sal-T by Sex

	Men						Women					
	Denominators, n		se	Mean Sal-T, pmol/L	Age-Adjusted Coefficients	(95% CI)	Denominators, n		se	Mean Sal-T, pmol/L	Age-Adjusted Ratios^b^	(95% CI)
	Unwt	Wt					Unwt	Wt				
All	1599	1886	3.33	223.49			2123	1899	0.86	37.09		
Any cardiac or vascular disease or hypertension					*P* = .0019						*P* = .6863	
No	1257	1548	3.75	235.47	—	—	1789	1625	0.95	37.99	1.00	—
Yes	342	338	4.92	168.6	−18.06	(−29.43, −6.68)	334	275	1.71	31.79	0.98	(0.88, 1.09)
Diabetes					*P* = .0932						*P* = .4689	
No	1482	1769	3.4	227.47	—	—	2036	1828	0.87	37.34	1.00	—
Yes	117	116	8.82	162.98	−15.55	(−33.71, 2.61)	87	71	2.54	30.67	0.93	(0.77, 1.13)
Currently taking medication for depression					*P* = .0428						*P* = .5670	
No	1513	1810	3.42	224.79	−	−	1930	1737	0.92	37.29	1.00	—
Yes	86	75	11.61	192.34	−24.56	(−48.33, −0.79)	193	163	2.11	34.99	0.97	(0.86, 1.09)
Depressive symptoms^a^					*P* = .7057						*P* = .7079	
No	1386	1644	3.41	223.84	—	—	1864	1646	0.81	37.13	1.00	—
Yes	176	186	11.25	228.68	−4.00	(−24.77, 16.77)	222	206	3.74	38.04	0.97	(0.85, 1.12)

Abbreviations: unwt, unweighted; wt, weighted.

a Participants were asked whether they had often been bothered by feeling down, depressed, or hopeless in hte past 2 weeks, and whether they had often been bothered by little interest or pleasure in doing things in the past 2 weeks, using a validated two-question patient health questionnaire (PHQ-2).

b Ratio of geometric means, obtained from exponentiated age-adjusted linear regression coefficients of log-transformed data for women.

## Discussion

### Summary of findings and comparison with other studies

This study is the first to show associations between Sal-T and health in a large national probability-sample survey of men and women, across a wide age range. In men, we found significant age-independent associations between lower Sal-T and higher BMI, poorer self-reported general health, mobility problems, longstanding illness, and comorbid conditions (cardiovascular disease, treatment for depression). The association between Sal-T and self-reported general health in men was attenuated after adjustment for BMI, suggesting that the relationship is at least partly explained by obesity. We found no associations between Sal-T and health factors in women, except smoking, with higher Sal-T among women who smoked.

The present finding of an age-related decline in Sal-T that persisted even after adjusting for health and demographic factors is consistent with previous serum studies that found not only serum total-T, but also serum free-T, declined across an age range in women (9) and men similar to Natsal-3 (24). Others, however, have argued that the age-related decline in serum total-T may entirely be explained by declining health (6). This inconsistency may partly be explained by the age-related increase in SHBG, which attenuates the age trend in total serum-T, but not serum free-T (5) or Sal-T.

Our findings of associations with health-related factors in men are largely consistent with evidence from serum studies. There is a large body of evidence that, in men, obesity is strongly associated with lower serum-T, independently of age (3). Associations have also been reported between lower serum-T and insulin resistance and diabetes, preclinical indicators for cardiovascular disease, cardiovascular events, physical frailty, and increased mortality (3, 5, 25, 26). Cross-sectional data cannot shed light on the direction of these relationships but longitudinal studies have found that obesity leads to decreases in T, and weight loss increases T levels in obese men (27), although there is also evidence of bidirectional associations (3). Longitudinal serum-T studies have shown that low T precedes cardiovascular events (25). The mechanisms of this association are unclear and may involve low T affecting several cardiovascular risk factors, central adiposity, and inflammation (28). Unlike most serum-T studies we did not find an age-adjusted association with self-reported diabetes for men with *P* = .0932 indicating at best weak evidence of an association, which did not persist after adjusting for other confounding factors including BMI. Although prevalence of diabetes in our sample was similar to national estimates (29), the absolute number of men with diabetes in our sample was relatively small, thus limiting our power to detect an association. However other research has shown that the apparent association between diabetes and low serum total-T, but not free-T, may be confounded by obesity and low SHBG, which is in line with our findings (30). We found no association with current depressive symptoms but did observe an association with treatment for depression. Little research exists on the effects of antidepressants on the hypothalamic-pituitary-gonadal axis, although one study has found higher Sal-T levels among men and women using selective serotonin reuptake inhibitors, which contrasts with our results (31).

We examined associations between Sal-T and several demographic factors to address potential confounding of the associations with health. A previous study found ethnic variation in T levels; however, we were unable to examine this due to small numbers of participants in ethnic minority groups. The findings of this study only partly concur with those from (generally smaller) studies that have reported lower T among men and women in established relationships (32) and parents, especially those actively involved in childcare (33, 34). We found associations with relationship status for men only, and no associations with parenthood for either men or women; however, our measures of parenthood capture a broad range of circumstances regarding children's age and parents' involvement in child rearing, which may explain this apparent discrepancy.

We found no association between Sal-T and smoking, alcohol consumption, or drug use in men. Previous serum-T studies in men have generally, although not always consistently, shown smoking to be associated with increased total-T, and studies of the associations with free-T have yielded mixed results (35). Smoking, via direct effects on liver function, increases SHBG levels, which may explain why total-T is increased in smokers yet in our study we did not find an association with Sal-T in men (35). The evidence regarding whether T is associated with alcohol consumption is also mixed (26, 36), and few studies have examined associations with other drugs.

Previous research about the health correlates of T in women has not only yielded inconsistent findings, but has largely been carried out within narrow age ranges, and using suboptimal measures of T (14). We found no evidence of associations between Sal-T and general health indicators, or specific conditions or medications in women. We did find a positive association with smoking, which is consistent with other studies (11, 37). We also found lower mean Sal-T among women who had taken HC, which can be explained by the combined effects of a direct inhibitory effect on ovarian androgen synthesis, an increase in SHBG concentration, and an inhibitory effect on adrenal androgen production (38). The association between Sal-T and smoking in women became nonsignificant after excluding women who had taken HC; however, the effect estimate was similar, so it is likely that this change in statistical significant is due to the reduction in sample size and resulting increase in confidence intervals, rather than a true difference between these groups of women.

Our findings regarding the different relationship with health and obesity for men and women may relate to the different sites of production and mechanisms of regulation. Among women, T is produced from the adrenal glands, regulated by adrenocorticotropic hormone, as well as the ovaries, regulated by gonadotropins, and there are menstrual cyclical fluctuations of ovarian steroids, whereas in men T is produced predominantly from the testes with only a very minor contribution from the adrenals. The mechanisms underlying the relationship between obesity and low T in men remain unclear, although the potential importance of circulating inflammatory cytokines from visceral adipose depots is gaining credence (39, 40).

### Strengths and limitations

A key strength of this study is the highly sensitive and specific Sal-T assay, enabling accurate measurement in women as well as men—although there is some systematic positive bias due to salivary protein binding among women (19)—and permitting measurement of T on a large-scale probability-sample survey across a wide age range. Although less invasive than serum, large-scale home-based collection of saliva has presented a number of challenges including coping with the diurnal variation in T levels, preventing contamination, ensuring prompt receipt at the laboratory before deterioration, and minimizing nonresponse bias. Considerable attention was paid to the development of protocols for sample collection, with extensive validation and piloting (20), yet although 71.0% of participants agreed to provide a sample, useable samples were received from only 45.0% of those invited, highlighting the challenge of obtaining self-collected and self-posted samples. This response rate is similar to community-based serum T studies (41, 42), and the response rate to the survey overall was similar to other major British social surveys. However, there were some systematic differences in the characteristics of those who returned a valid saliva sample, for example older participants were more likely to give a sample; therefore, to minimize potential nonresponse bias, both to the survey and to the saliva sample, we applied statistical weights during analysis (20).

The health data collected were self reported and are therefore reliant on knowledge of conditions and medications, and accurate reporting. This may particularly affect BMI based on self-reported height and weight, given that people tend to underestimate weight and overestimate height. However, previous studies have shown self-reported height and weight to be sufficient for examining associations in epidemiological studies (43). As a sexual health survey, only a limited number of questions about general health could be asked; therefore, we were unable to look at associations with some factors that may have been of interest such as frailty, osteoporosis, or sleep disturbance. We were also unable to measure preclinical disease indicators.

Only one sample was collected from each participant and so we were unable to take into account intra-individual variation in T levels, which may be particularly relevant for premenopausal women given that T varies throughout the menstrual cycle (44). However, some have argued that it is unnecessary to control for menstrual variation, given the relatively small effects compared with, for example, diurnal variation or individual differences (44). This is consistent with our earlier validation work in which we did not find significant within-individual differences when samples were taken at weekly and monthly intervals (17). A small number of men and women (n = 20 and n = 37, respectively) included in our analysis reported receiving cancer treatment in the past year, and as the nature of the cancer or treatment was unknown, this could have affected T levels for some. However, given the small numbers this is unlikely to have affected our findings overall.

### Implications for clinical research, policy, and additional research need

The findings presented in this paper provide crucial background for future research into the relationship between Sal-T and sexual function and sexual behavior, as well as being important in their own right for understanding associations between Sal-T and health factors. Our findings are broadly consistent with previous research using serum, and where differences exist it is not always clear whether these are due to differences in the measure used (serum total-T, free-T, or Sal-T), or due to other differences in the study population and design. Further observational research using reliable saliva T measurements linked to a broader range of clinical correlates would strengthen the evidence base in this respect.

There are concerns about inappropriate marketing and use of TRT for men, particularly in the United States (7). Our cross-sectional finding of lower Sal-T among men with poorer health does not imply causality, nor does it indicate treatment. Although longitudinal research has shown that low T precedes poor health outcomes (25) the benefits of TRT in the general population remain unclear. Further research is needed before conclusions can be reached regarding the nature of the relationship between T and ill health, and the risks and benefits of intervention.

Our finding of an independent age-related decline in Sal-T suggests that reproductive senescence in men, as in women, is not solely the consequence of poor health. The clinical significance of this merits further investigation using Sal-T as well as serum-T measurements. The application of Sal-T measurement in future research should make a significant contribution toward clarifying the role of low T in health and ageing in men and women.

## References

[1] Bhasin S Cunningham GR Hayes FJ Testosterone therapy in men with androgen deficiency syndromes: An Endocrine Society Clinical Practice Guideline. J Clin Endocrinol Metab. 2010;95(6):2536–2559. 20525905 10.1210/jc.2009-2354

[2] O'Connell MD Wu FC Androgen effects on skeletal muscle: Implications for the development and management of frailty. Asian J Androl. 2014;16(2):203–212. 24457838 10.4103/1008-682X.122581 PMC3955329

[3] Yeap BB Araujo AB Wittert GA Do low testosterone levels contribute to ill-health during male ageing? Crit Rev Clin Lab Sci. 2012;49(5–6):168–182. 23094995 10.3109/10408363.2012.725461

[4] Haring R Baumeister SE Völzke H Prospective inverse associations of sex hormone concentrations in men with biomarkers of inflammation and oxidative stress. J Androl. 2012;33(5):944–950. 22207707 10.2164/jandrol.111.015065

[5] Wu FC Tajar A Pye SR Hypothalamic-pituitary-testicular axis disruptions in older men are differentially linked to age and modifiable risk factors: The European Male Aging Study. J Clin Endocrinol Metab. 2008;93(7):2737–2745. 18270261 10.1210/jc.2007-1972

[6] Sartorius G Spasevska S Idan A Serum testosterone, dihydrotestosterone and estradiol concentrations in older men self-reporting very good health: The healthy man study. Clin Endocrinol. (Oxf. 2012) 77(5):755–763. 10.1111/j.1365-2265.2012.04432.x 22563890

[7] Nguyen CP Hirsch MS Moeny D Kaul S Mohamoud M Joffe HV Testosterone and “Age-Related Hypogonadism”—FDA Concerns. N Engl J Med. 2015;373(8):689–691. 26287846 10.1056/NEJMp1506632 PMC8905399

[8] Kamerow D Getting your “T” up. BMJ. 2014;348:g182–g182. 24412845 10.1136/bmj.g182

[9] Haring R Hannemann A John U Age-specific reference ranges for serum testosterone and androstenedione concentrations in women measured by liquid chromatography-tandem mass spectrometry. J Clin Endocrinol Metab. 2012;97(2):408–415. 22162468 10.1210/jc.2011-2134

[10] Davison SL Bell R Donath S Montalto JG Davis SR Androgen levels in adult females: Changes with age, menopause, and oophorectomy. J Clin Endocrinol Metab. 2005;90(7):3847–3853. 15827095 10.1210/jc.2005-0212

[11] Sowers MF Beebe JL McConnell D Randolph J Jannausch M Testosterone concentrations in women aged 25–50 years: Associations with lifestyle, body composition, and ovarian status. Am J Epidemiol. 2001;153(3):256–264. 11157413 10.1093/aje/153.3.256

[12] Sutton-Tyrrell K Wildman RP Matthews KA Sex hormone–binding globulin and the free androgen index are related to cardiovascular risk factors in multiethnic premenopausal and perimenopausal women enrolled in the Study of Women Across the Nation (SWAN). Circulation. 2005;111(10):1242–1249. 15769764 10.1161/01.CIR.0000157697.54255.CE

[13] Santoro N Torrens J Crawford S Correlates of circulating androgens in mid-life women: The Study of Women's Health Across the Nation. J Clin Endocrinol Metab. 2005;90(8):4836–4845. 15840738 10.1210/jc.2004-2063

[14] Wierman ME Basson R Davis SR Androgen therapy in Women: An Endocrine Society Clinical Practice Guideline. J Clin Endocrinol Metab. 2006;91(10):3697–3710. 17018650 10.1210/jc.2006-1121

[15] Vining RF McGinley RA Symons RG Hormones in saliva: Mode of entry and consequent implications for clinical interpretation. Clin Chem 1983;29(10):1752–1756. 6225566

[16] Macdonald PR Owen LJ Wu FC Macdowall W Keevil BG, NATSAL Team. A liquid chromatography–tandem mass spectrometry method for salivary testosterone with adult male reference interval determination. Clin Chem. 2011;57(5):774–775. 21300739 10.1373/clinchem.2010.154484

[17] Keevil BG MacDonald P Macdowall W Lee DM Wu FCW, the NATSAL Team. Salivary testosterone measurement by liquid chromatography tandem mass spectrometry in adult males and females. Ann Clin Biochem Int J Biochem Lab Med. 2014;51(3):368–378. 10.1177/0004563213506412 PMC5029560 24194586

[18] Keevil BG Fiers T Kaufman JM Sex hormone–binding globulin has no effect on salivary testosterone [published online ahead of print April 26, 2016]. Ann Clin Biochem. 10.1177/0004563216646800. 27117450

[19] Fiers T Delanghe J T'Sjoen G Van Caenegem E Wierckx K Kaufman JM A critical evaluation of salivary testosterone as a method for the assessment of serum testosterone. Steroids. 2014;86:5–9. 24793565 10.1016/j.steroids.2014.04.013

[20] Erens B Phelps A Clifton S National survey of sexual attitudes and lifestyles 3: Technical report. London: NatCen Social Research; 2013.

[21] Erens B Phelps A Clifton S Methodology of the third British National Survey of Sexual Attitudes and Lifestyles (Natsal-3). Sex Transm Infect. 2014;90(2):84–89. 24277881 10.1136/sextrans-2013-051359 PMC3933071

[22] Arroll B Khin N Kerse N Screening for depression in primary care with two verbally asked questions: Cross sectional study. BMJ. 2003;327(7424):1144–1146. 14615341 10.1136/bmj.327.7424.1144 PMC261815

[23] Gao J Bioanalytical method validation for studies on pharmacokinetics, bioavailability and bioequivalence: Highlights of the FDA's Guidance. Asian J Drug Metab Pharmacokinet. 2004;4(1):5–13.

[24] Stata Statistical Software: Release 13. College Station, TX: StatCorp; 2013.

[25] Khaw KT Dowsett M Folkerd E Endogenous testosterone and mortality due to all causes, cardiovascular disease, and cancer in men: European prospective investigation into cancer in Norfolk (EPIC-Norfolk) Prospective Population Study. Circulation. 2007;116(23):2694–2701. 18040028 10.1161/CIRCULATIONAHA.107.719005

[26] Allen NE Appleby PN Davey GK Key TJ Lifestyle and nutritional determinants of bioavailable androgens and related hormones in British men. Cancer Causes Control. 2002;13(4):353–363. 12074505 10.1023/a:1015238102830

[27] Camacho EM Huhtaniemi IT O'Neill TW Age-associated changes in hypothalamic-pituitary-testicular function in middle-aged and older men are modified by weight change and lifestyle factors: Longitudinal results from the European Male Ageing Study. Eur J Endocrinol. 2013;168(3):445–455. 23425925 10.1530/EJE-12-0890

[28] Jones TH Testosterone deficiency: A risk factor for cardiovascular disease? Trends Endocrinol Metab. 2010;21(8):496–503. 20381374 10.1016/j.tem.2010.03.002

[29] Craig R Mindell J Health Survey for England 2011. London, UK: The NHS Information Centre for health and social care; 2012 Available at: http://www.hscic.gov.uk/catalogue/PUB09300/HSE2011-Sum-bklet.pdf Accessed April 14, 2016.

[30] Antonio L Wu FCW O'Neill TW Low free testosterone is associated with hypogonadal signs and symptoms in men with normal total testosterone. J Clin Endocrinol Metab. 2016:jc.2015-4106. 10.1210/jc.2015-4106 26909800

[31] Giltay EJ Enter D Zitman FG Salivary testosterone: associations with depression, anxiety disorders, and antidepressant use in a large cohort study. J Psychosom Res. 2012;72(3):205–213. 22325700 10.1016/j.jpsychores.2011.11.014

[32] van Anders SM Goldey KL Testosterone and partnering are linked via relationship status for women and ‘relationship orientation’ for men. Horm Behav. 2010;58(5):820–826. 20727357 10.1016/j.yhbeh.2010.08.005

[33] Gettler LT McDade TW Feranil AB Kuzawa CW Longitudinal evidence that fatherhood decreases testosterone in human males. Proc Natl Acad Sci. 2011;108(39):16194–16199. 21911391 10.1073/pnas.1105403108 PMC3182719

[34] Barrett ES Tran V Thurston S Marriage and motherhood are associated with lower testosterone concentrations in women. Horm Behav. 2013;63(1):72–79. 23123222 10.1016/j.yhbeh.2012.10.012 PMC3540120

[35] Kapoor D Jones TH Smoking and hormones in health and endocrine disorders. Eur J Endocrinol. 2005;152(4):491–499. 15817903 10.1530/eje.1.01867

[36] Svartberg J Midtby M Bønaa KH Sundsfjord J Joakimsen RM Jorde R The associations of age, lifestyle factors and chronic disease with testosterone in men: the Tromsø Study. Eur J Endocrinol. 2003;149(2):145–152. 12887292 10.1530/eje.0.1490145

[37] Barbieri RL Sluss PM Powers RD Association of body mass index, age, and cigarette smoking with serum testosterone levels in cycling women undergoing in vitro fertilization. Fertil Steril. 2005;83(2):302–308. 15705366 10.1016/j.fertnstert.2004.07.956

[38] Zimmerman Y Eijkemans MJ Coelingh Bennink HJ Blankenstein MA Fauser BC The effect of combined oral contraception on testosterone levels in healthy women: A systematic review and meta-analysis. Hum Reprod Update. 2014;20(1):76–105. 24082040 10.1093/humupd/dmt038 PMC3845679

[39] Veldhuis J Yang R Roelfsema F Takahashi P Proinflammatory cytokine infusion attenuates LH's feedforward on testosterone secretion: Modulation by age. J Clin Endocrinol Metab. 2016;101(2):539–549. 26600270 10.1210/jc.2015-3611 PMC4880122

[40] Dhindsa S Batra M Kuhadiya N Dandona P Oestradiol concentrations are not elevated in obesity-associated hypogonadotrophic hypogonadism. Clin Endocrinol (Oxf). 2014; 80(3):464. 23647503 10.1111/cen.12236

[41] Feldman HA Longcope C Derby CA Age trends in the level of serum testosterone and other hormones in middle-aged men: Longitudinal results from the Massachusetts Male Aging Study. J Clin Endocrinol Metab. 2002;87(2):589–598. 11836290 10.1210/jcem.87.2.8201

[42] Lee DM O'Neill TW Pye SR The European Male Ageing Study (EMAS): Design, methods and recruitment. Int J Androl. 2009;32(1):11–24. 18328041 10.1111/j.1365-2605.2008.00879.x

[43] Spencer EA Appleby PN Davey GK Key TJ Validity of self-reported height and weight in 4808 EPIC-Oxford participants. Public Health Nutr. 2002;5(4):561–565. 12186665 10.1079/PHN2001322

[44] van Anders SM Goldey KL Bell SN Measurement of testosterone in human sexuality research: Methodological considerations. Arch Sex Behav. 2014;43(2):231–250. 23807216 10.1007/s10508-013-0123-z

